# Distinct modulation of microglial amyloid β phagocytosis and migration by neuropeptides^i^

**DOI:** 10.1186/1742-2094-7-61

**Published:** 2010-10-11

**Authors:** Sigal Fleisher-Berkovich, Talia Filipovich-Rimon, Sarit Ben-Shmuel, Claudia Hülsmann, Markus P Kummer, Michael T Heneka

**Affiliations:** 1 Faculty of Health Sciences, Dept. of Clinical Pharmacology, Ben-Gurion University of the Negev, Beer-Sheva, Israel; 2 Division of Clinical Neurosciences, Dept. of Neurology, University of Bonn Medical Center, Bonn, Germany

## Abstract

Microglial activation plays an integral role in the development and course of neurodegeneration. Although neuropeptides such as bradykinin (BK), somatostatin (SST), and endothelin (ET) are known to be important mediators of inflammation in the periphery, evidence of a similar function in brain is scarce. Using immunocytochemistry, we demonstrate the expression of receptors for BK (B1, B2 subtypes), ET (ETA, ETB subtypes) and SST (SST 2, 3, 4 subtypes) in primary microglia and microglial cell lines. Exposure of BV2 and N9, as well as primary microglial cells to BK or SST increased Aβ uptake in a concentration-dependent manner, whereas endothelin decreased Aβ uptake. This was caused by increased phagocytosis of Aβ since the rate of intracellular Aβ degradation remained unaffected. All neuropeptides increased chemotactic activity of microglia. In addition, BK reduced Aβ-induced expression of proinflammatory genes including iNOS and COX-2. ET decreased the Aβ-induced expression of monocyte chemoattractant protein 1 and interleukin-6. These results suggest that neuropeptides play an important role in chemotaxis and Aβ clearance and modulate the brain's response to neuroinflammatory processes.

## Background

Besides the well described pathological hallmarks, the deposition of the amyloid β peptide (Aβ) in fibrillar plaques and neurofibrillar tangles of tau protein, neuroinflammation has a prominent role during Alzheimer disease (AD) [1]. This is expressed by activated microglia and reactive astrocytes localized to amyloid plaques, and the release of biochemical markers, like cytokines, chemokines and nitric oxide, that are found to be increased in the brains of patients with AD[2]. Microglia, the main immune effector of the brain, are able to migrate to sites of Aβ deposition and to eliminate Aβ by phagocytosis upon activation by multiple receptors amongst the toll-like receptors (TLR2, TLR4), scavenger receptor A and CD36.

It is known that certain neuropeptides decline during aging before the onset of AD [3,4]. Neuropeptides belong to a family of signalling molecules released by neurons acting on cell surface receptors. Their role in inflammation has been widely studied in peripheral tissues [5,6], but their particular function on glial cells has not been studied in detail so far. There is growing evidence that neuropeptides are able to alter neurodegenerative processes during AD [7-10]. Strikingly, brain regions like the hippocampus and the cortex that are affected early during AD, are also the regions where neuropeptides like bradykinin and somatostatin, as well as their receptors, are prominently present [11-13].

Somatostains are cyclic peptides that are widely expressed throughout the brain where they act as neuromodulators [14]. Five G protein-coupled somatostatin receptors have been described (SSTR1-5), which are expressed in distinct yet overlapping patterns within the brain [15]. Of note, two of them, SSTR2 and SSTR4, are expressed in the hippocampus and cortex [16]. In AD, elimination of SST from the cortex and hippocampus is correlated to reduced cognitive function and memory impairment [10,17]. In addition, SSTRs show marked depletion in the AD brain [18-20].

Bradykinin (BK) is a short-lived nonapeptide generated from the kininogen precursors that is upregulated after lesions in the CNS [21]. Because of its attenuating effect on the secretion of pro-inflammatory cytokines from microglia BK has anti-inflammatory and thereby neuroprotective properties [21]. Like somatostatin, bradykinin binds to G protein-coupled receptor at the cell surface. Two subtypes of bradykinin receptor, B1 and B2, have been characterized so far. The expression of B2 bradykinin receptors has been confirmed in the brain stem, basal nuclei and cerebral cortex, whereas B1 bradykinin receptors are present in the entorhinal cortex, dentate gyrus, and pyramidal neurons of the hippocampus. Besides neurons and astrocytes, primary microglia have been shown to express BK receptors [22].

Another prominent neuropeptide system in the brain is the endothelin (ET) system. Endothelins are 21 amino acid long cyclic peptides. Currently there are three described isoforms (endothelin 1-3), that derive from precursor proteins by proteolytic processing [23]. They signal through two major receptor subtypes known as ETA and ETB receptors belonging to the superfamily of G-protein coupled receptor [23]. ET-1, ET-3 as well as ET receptors are expressed in glial and neuronal cells [23]. During AD it has been observed that endothelin-1 levels are decreased in the cerebrospinal fluid of AD patients [9] and its expression has been found to be elevated in frontal and occipital cortex in AD [7].

Since there are multiple indications that suggest an important role for neuropeptides in inflammation in general and in particular during the chronic inflammatory processes of AD, we investigated the impact of these signaling molecules on inflammatory core functions of microglial cells.

## Methods

### Primary microglial cell culture and microglial cells lines

Primary microglial murine cell cultures were prepared as previously described in detail (Hanisch et al., 2004). Briefly, mixed neuronal cultures were prepared from newborn mice and cultured in DMEM supplemented with 10% FCS and 100 U ml^-1 ^penicillin/streptomycin. Microglial cells were harvested by shake-off after 10-14 days of primary cultivation. Primary rat microglial cultures were obtained from the whole brain of newborn Wistar rats at 0-24 h of age. Mixed glial cells were cultured in High glucose (4.5 mg/ml) DMEM, supplemented with 10% FCS, 0.2 mM L-glutamine, 0.1 mg/ml streptomycin, and 0.2 U/ml penicillin was used as the culture medium. After 12 days, to isolate microglia, cells were shaken at 200 rpm for 1 h. The medium containing detached microglia was collected and the isolated microglia were reseeded and allowed to settle for 24 h [24]. BV2 and N9 cell lines were mantained in IMDM supplemented with 5% FCS and 100 U ml^-1 ^penicillin/streptomycin.

### Phagocytosis of FAM-labeled Aβ_1-42_

Microglial phagocytosis of aggregated FAM-labeled Aβ1-42 (FAM-Aβ) (Anaspec) was measured by plate based assay after incubation of Aβ at 37°C for three days. Cells were plated at 50000 cells in 100 μl in black 96 well plates. After 1 h Aβ was added to a final concentration of 500 nM and incubated for up to 4 h. Finally, the Aβ-containing medium was removed and extracellular Aβ was quenched with 100 μl 0.2% trypan blue in PBS pH 4.4 for 1 min. After aspiration fluorescence was measured at 485 nm excitation/535 nm emission using a Infinite 200 reader (Tecan). To normalize for cell numbers 100 μl 50 μg/ml Hoechst Dye 33342 in PBS was added, incubated for 30 min and the fluorescence measured at 360 nm excitation/465 nm emission. Additionally, microglial Aβ phagocytosis was verified by confocal microscopy. Confocal microscopy was performed using a BX61 microscope equipped with a disc spinning unit (Olympus).

### Assessment of microglial migration

Transwell 96 well permeable supports, containing 8 μm pore size polyester membrane, were used. Medium containing 10^-9^- 10^-7 ^M bradykinin (BK), endothelin-1 (ET) or somatostatin (SST) was added to the lower chamber, whereas cells, (10^6 ^cells/ml), were added to the top chamber and incubated for 3 h at 37°C. Cells that passed through the membrane were harvested from the lower part of the membrane using trypsin, and stained with calcein AM dye. The stained trypsinized cells were transferred to a black plate and read in a plate reader at 485 nm excitation, 520 nm emission.

### Immunocytochemistry of neuropeptide receptors

Cells were fixed in 4% paraformaldehyd at room temperature, washed twice with 100 mM HEPES pH 7, incubated in 1 μg/ml FC-block (Calbiochem), permeabilized with 0,1% Tx-100 in PBS for 10 min and non specific binding was blocked with 10% goat serum in PBS for 2 h. Sample were incubated overnight with first antibody in blocking solution, washed three times with PBS and the secondary antibody was added for 1 h in 10% goat serum/PBS. Finally the cells were washed three times in PBS, once in water and mounted with Immomount (Thermo) containing 5 μg/ml Hoechst 33342. Confocal microscopy was performed using BX61 equipped with a disc spinning unit (Olympus). Images were deconvoluted using the next neighbor algorithm (Cell^P, Olympus)

### Determination of microglial Aβ_1-42 _degradation

BV2 and N9 cells were incubated in a black 96-well plate (50,000 cells per well) and incubated for 15 h with bradykinin, endothelin or somatostatin. Then, FAM-labelled Aβ was added. After 30 min, all media were removed, cells mildly washed with warmed medium and subsequently fresh medium added for the indicated time points (0, 1, 2, 3 or 4 h). At each time-point media were removed and replaced by 0.2% trypan blue solution for 1 min in room temperature. Fluorescence detection of FAM-labelled Aβ_1-42 _was performed as described above. In order to confirm that cells were plated equally, cells were stained with Hoechst 33342.

### Western blot

BV2 cells were seeded onto a 6-well plate at a concentration of 2 × 10^6 ^cells per well and incubated in full medium for one hour. Then, medium was replaced by serum-free medium (SFM) in the absence or presence of the respective neuropeptide. After 15 h incubation, the cells were added with SFM or Aβ1-42 (at final concentration of 0.5 μM) and incubated for additional 4 h. After removal of SFM, cells were lysed in lysis buffer (20 mM Tris-Hcl pH 7.5, 0.5 mM EDTA, 0.5% Triton X-100 and 5 μM sodium vanadate) in the presence of protease inhibitors. Protein concentration was determined by bicinchoninic acid (BCA) assay kit (Thermo). Cell lysates were separated in 7.5% SDS-PAGE and blotted onto a nitrocellulose membrane (Biorad). Membranes were blocked for 90 min with 4% BSA and incubated overnight at 4°C with anti-NEP antibody (1:4000) (Abcam) or anti-IDE antibody (1:4000) (Calbiochem). After washing membranes were incubated for 90 min with the HRP-conjugated secondary antibody. Immunoreactivity was detected using enhanced chemiluminescence (Millipore) solution followed by exposure to X-ray film (Fuji medical X-ray film, FujiFilm). Semi-quantitative analysis was carried out using a computerized image analysis system (EZ Quant - Gel 2.2, EZQuant Biology Software Solutions). Protein load was normalized detecting β-actin (1:4000, Sigma).

### Real-Time Reverse Transcription-Polymerase Chain Reaction

Total RNA was prepared from the cells using RNeasy mini kit (Qiagen) and reversed transcribed (RT) into complementary DNA using high capacity cDNA reverse transcription kit (Applied Biosystems) according to the manufacturer's instructions. Real time qPCR was performed on a StepOnePlus Real-Time PCR System (Applied Biosystems) using TaqMan gene expression assay (Applied Biosystems). PCR was carried out in 20 μl with 1 μl of the RT product corresponding to 40 ng of total RNA, 0.2 μM of each primer and 10 μl of the master mix with the following temperature profile: 95°C for 10 min and 40 cycles of 95°C for 15 s and 60°C for 1 min. Amplification specificity was verified by melting curve analysis. mRNA expression values were normalized to the level of GAPDH expression. For primer details see Table 1.

### Statistical analysis

The immunohistochemical and densitometric data and were analyzed by 1-way, 2-way ANOVA and Tukey's's post hoc test (GraphPad Prism 5 or SPSS 17).

## Results

**Figure 1:**
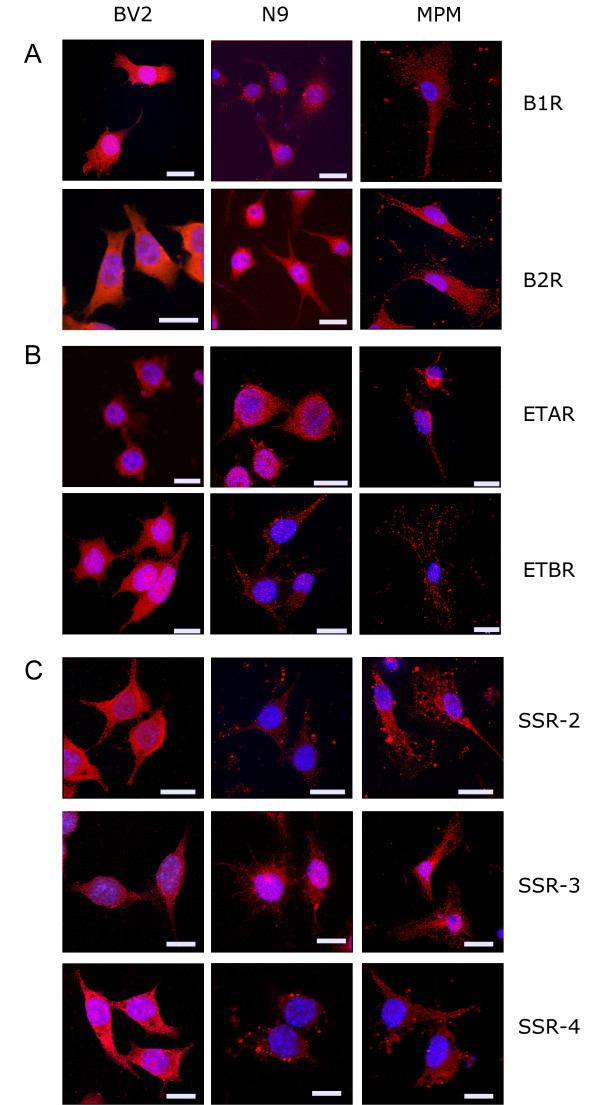
**Immunocytochemical analysis of neuropeptides receptors expression**. BV2, N9, and murine primary microglia cells were immunostained with specific primary antibodies against bradykinin receptors 1 and 2 (B1R, B2R), endothelin A receptor (ETAR), endothelin B receptor (ETBR), somatostatin receptors 2,3,4 (SSTR2-4). Nuclei were visualized using Hoechst 33342.

In order to assess whether neuropeptides are involved in the regulation of microglial functions, we first assessed the presence of the neuropeptide receptors on the cells lines and murine primary cells utilized in the following experiments. BV2, N9 and primary murine microglia expressed the bradykinin receptors 1 and 2, the endothelin receptor A and B as well as the somatostatin receptors 2,3 and 4 (Figure 1). These immunocytochemical experiments document that indeed all the above described neuropeptide receptors can be detected on the microglial cell surface and thus may be involved in mediating the modulating effects on Aβ_1-42 _phagocytosis and migration described below.

**Figure 2:**
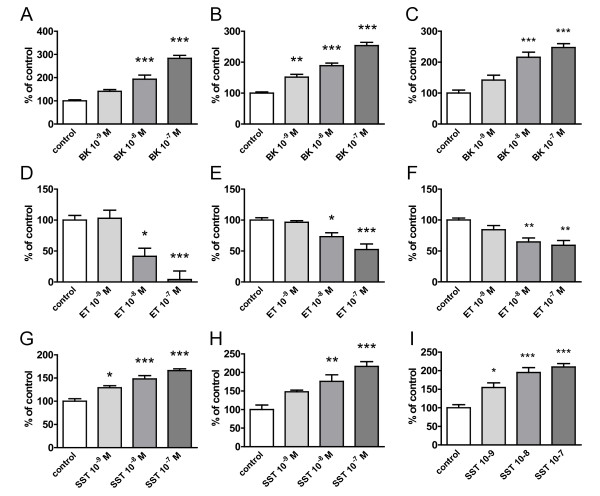
**Neuropeptides modulate microglial Aβ_1-42 _phagocytosis**. FAM-Aβ_1-42 _phagocytosis was examined in BV2 cells (A, D, G), N9 cells (B, E, H) and primary rat microglia (C, F, I). Cells were incubated for 15 h with bradykinin (BK) (A-C), endothelin (ET) (D-F) or somatostatin (SST) (G-I) at concentrations of 10^-9^-10^-7 ^M. Thereafter, 0.5 μM of FAM-labelled Aβ was added and phagocytosis assessed after 4 h. (n = 4 +/- SEM; one-way ANOVA followed by Tukey's post test, * P < 0.05 vs. control, ** P < 0.01 vs. control. *** P < 0.001 vs. control).

Microglial Aβ_1-42 _phagocytosis was assessed in two microglial cell lines, BV2 and N9 cells as well as primary rat microglia which were incubated with either bradykinin, endothelin or somatostatin for 15 h and subsequently exposed to aggregated and FAM-labelled Aβ_1-42_. While concentrations of 10^-9^-10^-7 ^M of bradykinin and somatostatin increased Aβ_1-42 _phagocytosis concentration-dependently, endothelin showed the opposite effect (Figure 2). There was no principle difference in quantity of quality regarding the observed effects in microglial cell lines compared to primary rat microglia, suggesting that neuropeptide effects can be studied reliably in the two immortalized cell lines which were subsequently used.

**Figure 3:**
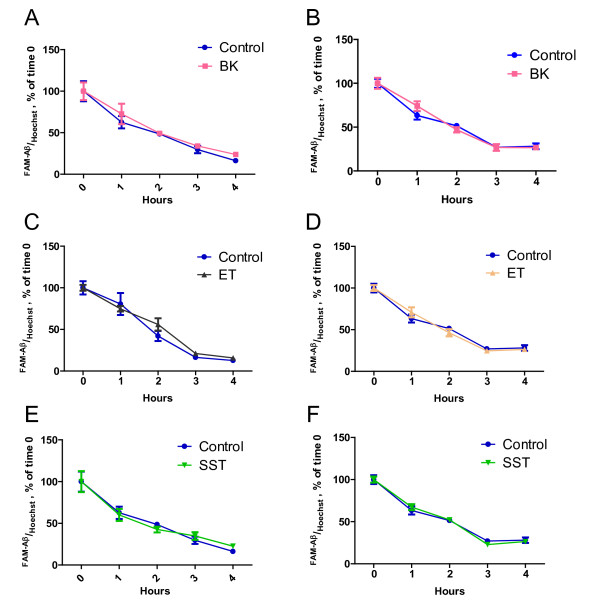
**Microglial Aβ_1-42 _degradation is not affected by neuropeptides**. BV2 and N9 cells were incubated for 15 h with bradykinin (BK) (A, B) or endothelin (ET) (C, D) or somatostatin (SST) (E, F). Then, FAM-labelled Aβ was added and phagocytosis of Aβ_1-42 _was allowed for 30 min. Thereafter all media were removed and fresh medium was added for the indicated time periods. In order to confirm that cells were plated equally, cells were stained with Hoechst 33342 (n = 4 +/- SEM).

**Figure 4:**
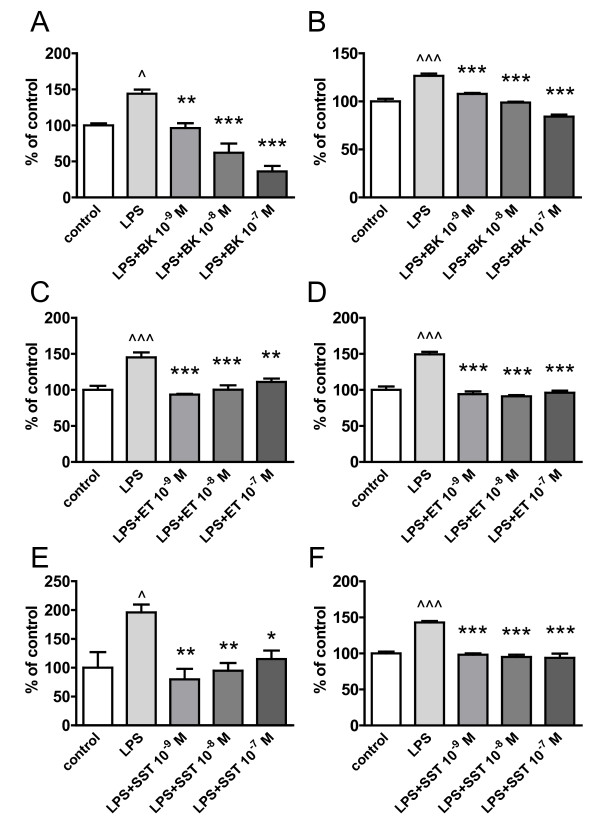
**Immunostimulation with LPS affects neuropeptide regulation of microglial Aβ_1-42 _phagocytosis**. BV2 (A, C, E) and N9 cells (B, D, F) were incubated for 15 h with LPS alone (0.1 μg/ml) or in the presence of bradykinin (BK) (A, B), endothelin (ET) (C, D) or somatostatin (SST) (E, F) at concentrations of 10^-9^-10^-7 ^M. Afterwards cells were incubated with FAM-Aβ_1-42 _for 4 h and the amount of phagocytosed FAM-Aβ_1-42 _was measured spectrometrically and normalized to untreated control cells (n = 3 +/- SEM; one-way ANOVA followed by Tukey's post test, ^ P < 0.05 vs. control, ^^^ P < 0.001 vs. control. * P < 0.05 vs. LPS ** P < 0.01 vs. LPS. *** P < 0.001 vs. LPS).

Principally, the observed effects of neuropeptides on microglial Aβ_1-42 _uptake after 4 h could also have resulted from an impairment of Aβ_1-42 _degradation within microglia instead of an increase of phagocytosis. We therefore tested whether neuropeptides would influence the Aβ_1-42 _content of microglia over time after an initial short exposure of cells to the peptide for only 30 min. Importantly, microglial cells did phagocyte Aβ_1-42 _within the first 30 min. However, neither bradykinin, endothelin nor somatostatin affected the microglial Aβ content which showed a progressive decline over 4 h, consistent with the hypothesis of an unaffected intracellular degradation (Figure 3). None of the neuropeptides accelerated the decomposition of Aβ_1-42 _within microglia. Together, these experiments validate that neuropeptides positively modulate the phagocytosis of Aβ but do not affect its intracellular degradation. Next it was analyzed whether the observed modulation of Aβ_1-42 _phagocytosis was preserved when cells were simultaneously pre-exposed to an immune challenge. Therefore, BV2 and N9 cells were incubated for 15 h either with lipopolysaccharide (LPS) alone (0.1 μg/ml) or together with bradykinin, endothelin or somatostatin. LPS stimulation alone increased the phagocytosis of Aβ_1-42 _(Figure 4). In contrast to the previously observed stimulatory effect of bradykinin, costimulation of microglial cells with LPS lead to a concentration-dependent decrease of bradykinin stimulated Aβ_1-42 _phagocytosis (Figure 4A). LPS also abolished the attenuating effect of endothelin or the enhancing modulation of somatostatin that were observed under non-inflammatory conditions. However, all neuropeptides impaired the LPS-induced increase of Aβ_1-42 _phagocytosis in microglial cells.

**Figure 5:**
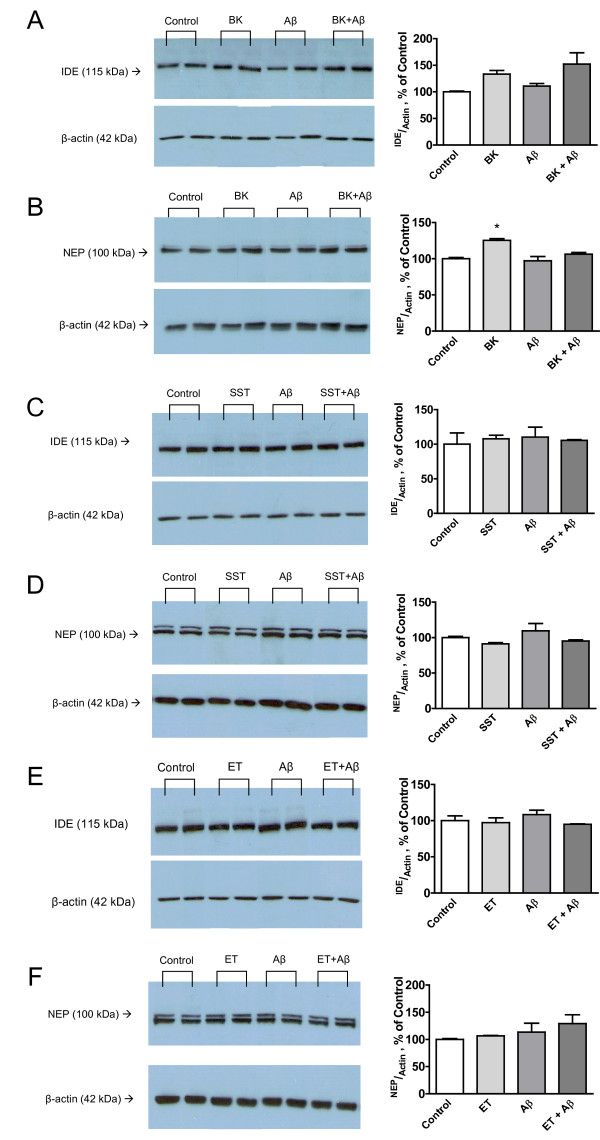
**Expression of neprilysin (NEP) and insulin-degrading enzyme (IDE) in BV2 cells**. Cells were incubated with bradykinin (BK) (A, B), somatostatin (SST) (C, D) or endothelin (ET) (E, F) at concentrations of 10^-7 ^M each. Then, Aβ_1-42 _(0.5 μM) was added and the cells incubated for additional 4 h. Western blot analysis was performed using whole cell lysates and antibodies directed against NEP (B, D, F), IDE (A, C, E) and β-actin. Representative gels of three independent experiments (n = 3 +/- SEM; one-way ANOVA followed by Tukey's post test, *P < 0.05).

Nevertheless, the possible effect of neuropeptides on the expression of two major Aβ degrading activities, namely, the insulin degrading enzyme (IDE) and neprylisin (NEP), was investigated under control conditions and in the presence of Aβ_1-42 _(Figure 5). While IDE was not found to be influenced by any of the conditions investigated, bradykinin increased the NEP under control conditions (Figure 5B). This effect, however, was abolished when cells were coincubated with Aβ_1-42_.

**Figure 6:**
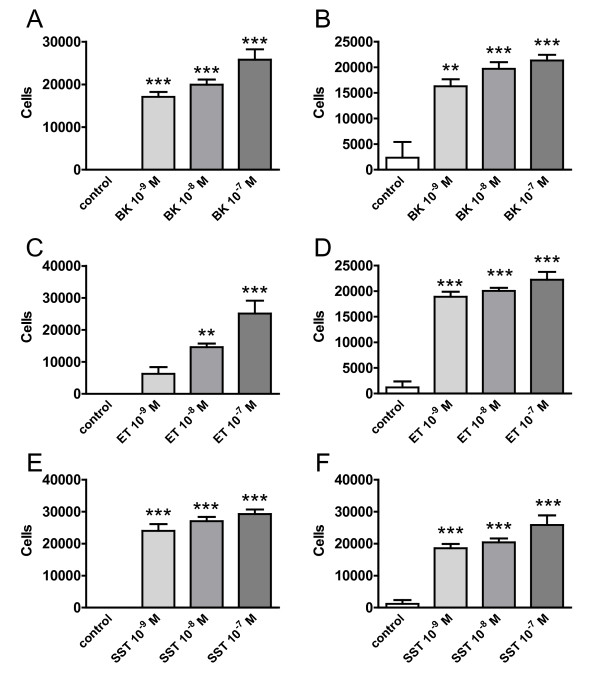
**Neuropeptides positively regulate microglial migration**. The effect of neuropeptides on migration of BV2 (A, C, E) and N9 (B, D, F) cells were analyzed in a transwell assay after 3 h incubation with increasing concentrations of BK (A, B), SST (C, D) or ET (E, F). Cells that passed through the membrane were harvested, stained with calcein AM dye, and fluorescence was read at 485 nm excitation and 520 nm emission. (n = 4 +/- SEM; one-way ANOVA followed by Tukey's post test. ** P < 0.01 vs. control. *** P < 0.001 vs. control.). Similar results were obtained in 2 additional experiments for BV2 and N9 cells.

While phagocytosis of Aβ was differentially regulated by neuropeptides, all neuropeptides investigated increased the capacity of microglia to migrate in a more or less concentration-dependent fashion (Figure 6), reaching indeed very strong effects already at the lowest concentrations.

**Figure 7:**
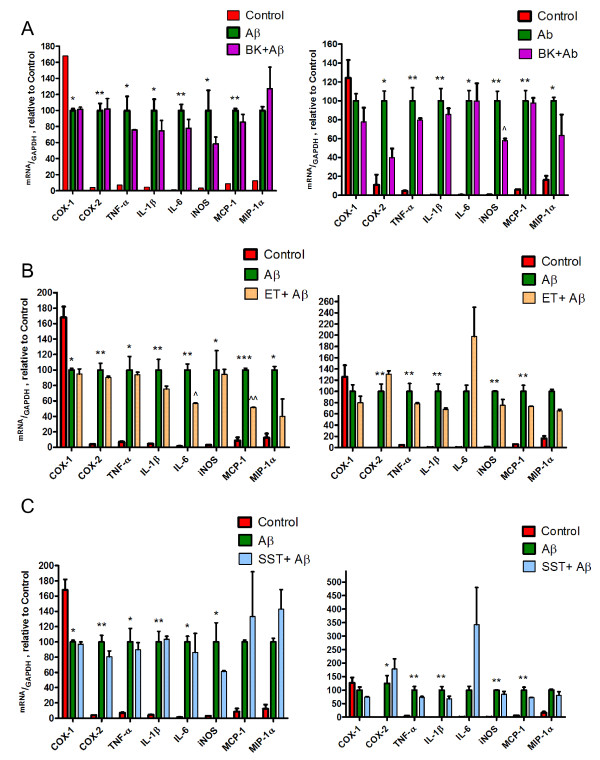
**Effect of neuropeptides on inflammatory genes expression**. BV2 (left column) and N9 (right column) cells were incubated with bradykinin (BK) (A), endothelin (ET) (B) or somatostatin (SST) (C) for 15 h at the concentration of 10^-7 ^M. Then, microglia were exposed to Aβ_1-42 _(1 μM) and cells harvested after 4 h. At the end of the experiment, total RNA was extracted and reverse transcribed. Finally, Real time qPCR was performed. mRNA expression was normalized to GAPDH. Similar results were obtained in additional experiment for BV2 and N9 cells (n = 2+/-SEM performed in triplicates; two-way ANOVA followed by Bonferroni multiple comparison. * P < 0.05 vs. control. ** P < 0.01 vs. control. *** P < 0.001 vs. control. ^ P < 0.05 vs. Aβ, ^^ P < 0.01 vs. Aβ).

To assess whether Aβ_1-42_-induced inflammatory gene transcription is being influenced by neuropeptides Aβ_1-42_-stimulated microglial BV2 and N9 cells were incubated with either bradykinin, somatostatin or endothelin. While exposure of cells to Aβ_1-42 _alone constantly decreased the expression of cyclooxygenase-1, all other targets investigated including cyclooxygenase-2, tumor necrosis factor-α, interleukin-1β, interleukin-6, inducible nitric oxide synthase (iNOS), monocyte chemotactic protein-1 and macrophage inflammatory protein-1α were robustly increased (Figure 7). Of the latter, only a few targets were negatively regulated by neuropeptides. Thus, bradykinin reduced iNOS gene transcription in N9 cells (Figure 7A), whereas this phenomenon did not reach the level of statistical significance in BV2 cells. In BV2 cells however, endothelin decreased IL-6 and MCP-1 gene transcription (Figure 7B), a results which was not reproduced in N9 microglial cells. Somatostatin did not affect inflammatory gene transcription of the above targets. In summary this analysis may indicate that the analyzed neuropeptides are no major regulators of inflammatory gene transcription and hence the observed effects on Aβ_1-42 _phagocytosis and migration are not influenced by inflammatory mediators.

## Discussion

Microglial cells represent the brain's innate immune system and are actively involved in maintaining parenchymal homeostasis through active tissue scanning and removal of debris by phagocytosis [25]. In neurodegenerative diseases, activation of microglia leads to an alteration of these physiological functions, which in turn may contribute to disease progression and neuronal death. Next to cytokines and chemokines, neurotransmitters including norepinephrine, glutamate and acetylcholine can modulate the inflammatory action of microglia and influence their functions [26,27]. Thus, neuronal activity and release of these factors may determine the activation status and finally the conditions whether microglia turn towards a beneficial or detrimental phenotype. In AD, various neurotransmitter systems are compromised and may thereby impair microglial functions in response to Aβ deposition including phagocytosis and migration [28]. Impaired microglial phagocytosis of Aβ, however, may directly contribute to disease progression and neuronal dysfunction.

The presence of neuropeptide receptors on microglia suggests that these factors may be actively involved in the regulation of microglial functions [22,29,30]. After confirming the expression of bradykinin, endothelin and somatostatin receptors on primary murine microglia as well as on the microglial cell lines, the capacity of microglia to phagocyte fibrillar Aβ after preincubation with increasing concentrations of either one of the respective neuropeptides was studied. Of note, the overall levels of the peptides are low in the normal rodent and human brain. In particular, the basal levels of endothelins have been found to be 0.37 × 10^-9 ^M in human cerebrospinal fluid [31], but undergo a massive upregulation upon brain injury [32,33]. Further, the release of somatostatin from incubated rat hypothalamus has been determined to be 3 × 10^-9 ^M [34]. Finally, the exact levels of brain bradykinin are unknown. Nevertheless, the literature is unanimous in that bradykinin, used at 10^-7 ^M and higher, stimulates glial inflammation [35]. We therefore used these peptides in the range from 10^-9 ^to 10^-7 ^M, corresponding to physiological as well as 10 to 100 fold increased concentrations.

While bradykinin and somatostatin increased microglial Aβ phagocytosis in a concentration-dependent manner, endothelin showed the opposite action, suggesting a distinct regulation of Aβ uptake by neuropeptides. Importantly, none of the investigated peptides altered the intracellular degradation of Aβ thus confirming that the observed differences are solely due to increased uptake by microglia. While there is no previous information on the modulation of microglial phagocytosis by these substances, in general a positive regulation of phagocytosis by bradykinin had been reported in peripheral leucocytes after intravenous application of this neuropeptide in rabbits [36]. In contrast, somatostatin had been found to suppress the phagocytic capacity of macrophages for Leishmania major parasites [37]. Together, these data suggest that the neuropeptides do not generally up- or downregulate phagocytosis, but the later action strongly involves target-specific mechanisms.

Since somatostatin is reduced in AD brain and in CSF of AD patients [38,39], one can speculate that this reduction contributes to Aβ deposition in the AD brain through impaired microglial phagocytosis along with the previously described negative influence on Aβ degradation by neprilysin [40]. Likewise, the increase of endothelin expression observed in AD frontal and occipital cortex could compromise the effective clearance of the brain from Aβ by reducing microglial phagocytosis [7].

Interestingly, immunostimulation of microglia with bacterial lipopolysaccharide (LPS) almost completely abolished the regulatory effects of neuropeptides on microglial Aβ phagocytosis. Systemic bacterial infection has been reported to aggravate the clinical symptoms of AD patients [41], a phenomenon which may be explained by several mechanisms. However, extrapolating the findings of this study, it can be hypothesized that an additional immune challenge, in this case by LPS, leads to the reduction of beneficial microglial functions evoked neuropeptides such as bradykinin and somatostatin -even if those are decreased in AD brains as stated above, since the increased Aβ uptake observed in response to these neuropeptides is compromised by pre-exposure to LPS.

Since in the CNS migration of microglial towards inflammatory lesion sites plays an important role in resolving neuroinflammation and restoring local homeostasis, the effect of the three neuropeptides on migration were investigated. In contrast to the divergent effects on phagocytosis, all three neuropeptides collectively increased microglial migration. Bradykinin has been shown before to stimulate microglial migration in a concentration- and time-dependent manner and seems to involve the bradykinin 1 receptor [42], however no positive regulation on migration in general, but inhibition of Leishmania major promastigote-induced macrophage migration had been reported previously for somatostatin [43].

Once phagocyted, microglial cells are able to degrade Aβ fibrils [44]. Several enzymatic systems have been described to be involved in this process, but the detailed mechanisms underlying phagolysosomal decomposition have not been elucidated. Both neprilysin (NEP) and insulin degrading enzyme (IDE) play the pivotal role for Aβ degradation in the brain [45]. While NEP is presumably acting within a cell, IDE is also being secreted and involved in extracellular Aβ degradation [46]. To test whether neuropeptides are also influencing the generation of these systems, in the absence or presence of Aβ, microglial expression of these enzymes was detected. In the case of Bradykinin there was a trend to increased levels of IDE, however this effect did not reach the level of statistical significance. Of note, the same neuropeptide increased microglial NEP levels, an effect that disappeared when cells were challenged with Aβ. The latter phenomenon suggests that Aβ has a negative regulatory function on NEP levels. The two other neuropeptides tested did not influence either NEP or IDE levels suggesting that this effect is specific for bradykinin. Since previous studies suggested an anti-inflammatory role of neuropeptides, in particular of bradykinin [47], we analyzed the regulation of inflammatory gene mRNA levels in response to Aβ stimulation and neuropeptide incubation. Under all conditions, incubation of microglial cells with Aβ strongly upregulated inflammatory gene transcription including tumor necrosis factor α, interleukin-1β, interleukin-6, inducible nitric oxide synthase (iNOS), monocyte chemoattractant protein-1, macrophage inflammatory protein-1α, cyclooxygenase-2, while cycloxygenase-1 was downregulated by the identical treatment. However, a significant decrease of inflammatory mRNA levels was only observed in N9 cells, where bradykinin decreased iNOS and in the BV2 cell line, where IL-6 and MCP-1 were negatively controlled by endothelin. It seems possible that the chosen time point have not allowed for the detection of stronger regulatory effects, however, it still suggests that the observed neuropeptide regulation of microglia function is a direct effect and not secondary to chemo- or cytokine regulation.

## Conclusion

Together our data suggest that neuropeptides may be critically involved in the modulation and guidance of microglial functions relevant for AD. Since these neuropeptides are generated and secreted from neurons, the latter may be intimately involved in immune cell regulation and modification of the neuroinflammatory component of AD. Future studies are needed to unravel the intracellular signaling pathways involved and to verify these data in appropriate animal models.

## Competing interests

The authors declare that they have no competing interests.

## Authors' contributions

SF designed the study, analyzed data and drafted the manuscript. TF and SB carried out the phagocytosis assay, the migration assays, the mRNA expression analysis and the Western blotting experiments. CH carried out phagocytosis assays and did immunocytochemical stainings. MPK did the immunocytochemical analysis, designed the study and drafted the manuscript. MTH conceived and supervised the study and drafted the manuscript. All authors read and approved the final manuscript.
